# Health belief model based evaluation of school health education programme for injury prevention among high school students in the community context

**DOI:** 10.1186/1471-2458-14-26

**Published:** 2014-01-10

**Authors:** Zhi-Juan Cao, Yue Chen, Shu-Mei Wang

**Affiliations:** 1 School of Public Health, “Key Laboratory of Public Health Safety, Ministry of Education”, Fudan University, 130 Dong’ an Road, Xuhui District, Shanghai, China; 2 Department of Epidemiology and Community Medicine, University of Ottawa, 451 Smyth Road Ontario, K1H 8 M5 Ottawa, Canada

## Abstract

**Background:**

Although multifaceted community-based programmes have been widely developed, there remains a paucity of evaluation of the effectiveness of multifaceted injury prevention programmes implemented in different settings in the community context. This study was to provide information for the evaluation of community-based health education programmes of injury prevention among high school students.

**Methods:**

The pre-intervention survey was conducted in November 2009. Health belief model (HBM) based health education for injury prevention started in January 2010 and stopped in the end of 2011 among high school students in the community context in Shanghai, China. A post-intervention survey was conducted six weeks after the completion of intervention. Injury-related health belief indicators were captured by a short questionnaire before and after the intervention. Health belief scores were calculated and compared using the simple sum score (SSS) method and the confirmatory factor analysis weighted score (CFAWS) method, respectively.

**Results:**

The average reliability coefficient for the questionnaire was 0.89. The factor structure of HBM was given and the data fit HBM in the confirmatory factor analysis (CFA) very well. The result of CFA showed that Perceived Benefits of Taking Action (BEN) and Perceived Seriousness (SER) had the greatest impact on the health belief, Perceived Susceptibility (SUS) and Cues to Action (CTA) were the second and third most important components of HBM respectively. Barriers to Taking Action (BAR) had no notable impact on HBM. The standardized path coefficient was only 0.35, with only a small impact on CTA. The health belief score was significantly higher after intervention (*p* < 0.001), which was similar in the CFAWS method and in the SSS method. However, the 95% confidential interval in the CFAWS method was narrower than that in the SSS method.

**Conclusions:**

The results of CFA provide further empirical support for the HBM in injury intervention. The CFAWS method can be used to calculate the health belief scores and evaluate the injury related intervention. The community-based school health education might improve injury-related health belief among high school students; however, this preliminary observation needs to be confirmed in further research.

## Background

Injury is a significant cause of death and morbidity among children from the age of one [1] and becomes the leading cause of death among children aged 10 to 19 years worldwide [2]. Unintentional injuries account for approximately 830 000 deaths each year among children. Road traffic injuries and drowning are the two leading causes of death, and rates are particularly high among children in poorer countries or poor neighborhoods within rich countries [3]. However, death represents just a small proportion of the injury burden, whereas nonfatal health outcomes represent a large component of the injury burden, including life-long disability, significant psychological trauma, and subsequent financial loss [4].

During the last 25 years, multifaceted community-based programmes have become an important approach to promote health and prevent injuries [5,6]. This approach emphasizes the importance of community member participation and multidisciplinary collaboration among local organizations [5,7]. Health education is the indispensable content of a multifaceted community-based injury intervention programme and is effective for injury control among pupils and middle school students [8]. Community-based health education is different from school-based health education; it can not only use more resources, including human resources, material resources and financial resources, but also gain support from administrative departments and communities. Community-based health education includes three levels of involvement. Community, school and family make a combined effort to provide health education to students.

The Health Belief Model (HBM) originated in 1950s as a systematic method to explain and predict preventive health behavior [9-11]. It focuses on two aspects of health behavior: threat perception and behavioral evaluation [12]. In terms of injury prevention, threat perception includes two components, susceptibility to an injury and anticipated severity of the consequences of an injury. Behavioral evaluation consists of two distinct sets of beliefs: those related to barriers to change injury related risk behaviors and those concerning benefits. In addition to threat perception and behavioral evaluation, “cues to action” component was also included in the HBM. “Cues to action” refers to triggers to change injury related risk behaviors [13].

**Figure 1:**
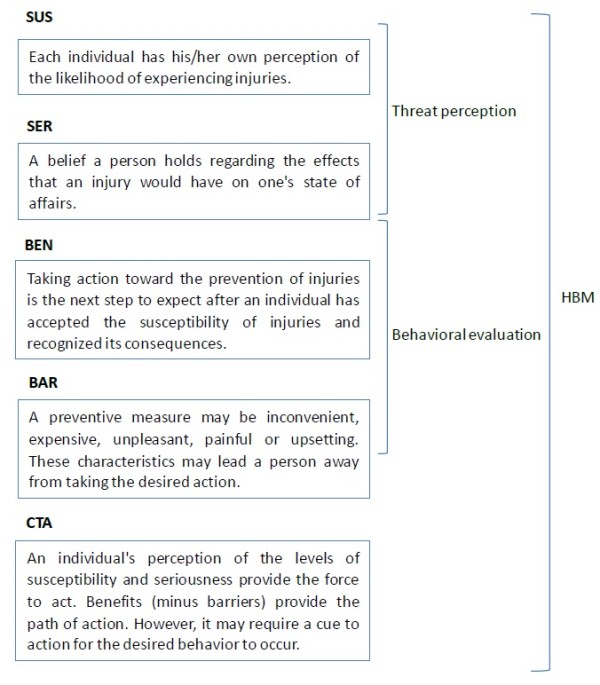
HBM components.

HBM are composed by 5 factors [11,12], the explanation of each factor concerning injury prevention is given below (Figure 1):

Perceived Susceptibility (SUS) - Each individual has his/her own perception of the likelihood of experiencing injuries. Individuals vary widely in their perception of susceptibility to injuries. Those at low end of the extreme deny the possibility of experiencing injuries. Individuals in a moderate category admit to a statistical possibility of injuries susceptibility. Those individuals at the high extreme of susceptibility feel there are real dangers that they will experience injuries.

Perceived Seriousness (SER) - A belief a person holds regarding the effects that injuries would have on one’s state of affairs. These effects can be considered from the point of view of the difficulties that injuries would create, for instance, pain and discomfort, loss of work time, financial burdens, difficulties with family, and susceptibility to future conditions. It is important to include these emotional and financial burdens when considering the seriousness of injuries.

Perceived Benefits of Taking Action (BEN) - Taking action toward the prevention of injuries is the next step to expect after an individual has accepted the susceptibility of injuries and recognized its consequences. The direction of action that a person chooses will be influenced by the beliefs regarding the action.

Barriers to Taking Action (BAR) - However, action may not take place, even though an individual may believe that the benefits to taking action are effective. This may be due to barriers. A preventive measure may be inconvenient, expensive, unpleasant, painful or upsetting. These characteristics may lead a person away from taking the desired action.

Cues to Action (CTA) - An individual’s perception of the levels of susceptibility and seriousness provide the force to act. Benefits (minus barriers) provide the path of action. However, it may require a cue to action for the desired behavior to occur. This cue may be internal or external.

Health educations based on the HBM have been shown to be effective in promoting a range of behavior changes [14-17]. Systematic reviews have supported the central tenets of the HBM [14,18,19]. The HBM suggests that changes in behavior result from changes in the putative social-cognitive determinants of behavior; thus, interventions should target these changes [20].

Evaluation of interventions is an important aspect of injury prevention, and statistical modelling plays a major part in large-scale evaluations [21]. The health belief evaluation has often involved comparisons of Simple Sum Score (SSS) method between pre-intervention and post-intervention. The SSS method gives each item an equal weight in the final score, which may lead to health belief score be made up of items with inefficient scoring weights [22]. However, instead of being measured directly, the level of health belief could only be shown through indirectly measurable variable, or latent variable. In the HBM, the level of five health beliefs (SUS, SER, BEN, BAR, CTA) cannot be directly measured, but can be reflected by other variables, which can be measured directly. These unobservable concepts must be quantified and measured by combining several relevant pieces of information. Measurement is frequently carried out through a scale administered to a study participant, which is summed to produce a score. From a statistical perspective, the goal of measurement is often to combine important pieces of information in a way that thoroughly describes an unobservable construct [23].

In order to make up for the deficiency of SSS method, a quasi-latent-variable-score method called Confirmatory Factor Analysis Weighted Score (CFAWS) method was used to calculate the weighted injury-related health belief score (WHBS) among high school students. The CFAWS method separates the variance among a set of observed variables into variance due to a common factor (latent variable), and variance due to the individual observed variables, and allows researchers to assign unequal weight to each item based on its degree of association with injury-related health belief [18].

Injury-related health belief scores were compared between pre-intervention and post-intervention through the SSS method and the CFAWS method to investigate whether the community-based health education can improve students’ health belief. In addition, this study used confirmatory factor analysis (CFA) to test the reliability and validity of the questionnaire and the HBM for injury intervention.

## Method

### Measurement instrument development and assessment

Following an extensive review of literatures and consultation with researchers and health professionals in this aspect, a short and easily self-administered questionnaire was designed to capture health belief indicators related to major injury types among high school students. The questionnaire was composed of five parts (22 items) according to the HBM: 5 indicators for SUS; 4 indicators for SER; 5 indicators for BEN; 4 indicators for BAR; and 4 indicators for CTA. The questionnaire was developed by modifying and expanding on the operational indicators used in earlier HBM studies [24] (see Table 1 for item mapping). Each item was rated on a 5-point scale using anchors between 1 and 5 (1 = strongly disagree, 2 = disagree, 3 = neutral, 4 = agree, 5 = strongly agree). Most items were related to traffic health belief and sports health belief because traffic and sports were the top two causes for injuries in this community according to an injury survey. Each item in SUS, SER, BEN, CTA gained a positive value 1 to 5, while items in BAR gained negative values of the original −1 to −5.

**Table 1:** Item mapping for HBM and total effect of health belief on each item

**HBM components**	**IN**	**Items**	**TE**
SUS	1	Climbing over road isolation barriers can easily lead to traffic injury	0.57
	2	Playing in the middle of the road can easily lead to traffic injury	0.57
	3	Cycling on the road with a passenger or without hands on the handlebars can easily lead to traffic injury	0.66
	4	Cycling on the road while racing other bikes or motorcycles can easily lead to traffic injury	0.66
	5	Travelling with a drunk driver of a car or motorcycle can easily lead to traffic injury	0.63
SER	6	A traffic injury can lead to scratches, muscle injury, cerebral concussion, even disability	0.73
	7	A fall can lead to strain or fracture	0.74
	8	Doing sports without protection (such as a kneelet or helmet) can lead to severe injury	0.71
	9	Burns and scalds can lead to a scar or even disability or death	0.61
BEN	10	Driving with seat belt fastened and cycling with a helmet can avoid traffic injury	0.72
	11	Doing prep before participating in sports can effectively avoid sports injury	0.78
	12	Quickly bending the head, touching the ground with shoulders and back and rolling on the ground can avoid injury when falling	0.74
	13	Doing sports with protection (such as a kneelet or helmet) can prevent injury	0.79
	14	Food safety can effectively avoid food poisoning	0.75
BRA	15	Hard to get used to fastening the seat belt while driving or to put on a helmet while driving a moped	−0.14
	16	Hard to get used to putting on a helmet while cycling	−0.14
	17	Hard to do prep before sport	−0.16
	18	Hard to do sports with protection (such as a kneelet or helmet)	−0.16
CTA	19	Injury prevention advertisements on TV have huge influence on you	0.48
	20	Injury prevention advertisements on the news and in magazines have huge influence on you	0.50
	21	Injury to friends or family has huge influence on you	0.53
	22	Friends or family opinion on injury have huge influence on you	0.54

The questionnaire was evaluated by a panel, which consisted of 8 people with expertise in epidemiology, injury prevention mapping, health education, and children and adolescent health, for its content validity and clarity. Pre-test was conducted among 100 students to test whether the questionnaire formulated each item clearly and gave prominence to the key points, and then the inappropriate items were modified to make sure every student understood the questions correctly. Age and gender were also recorded.

### Confirmatory factor analysis of the HBM structure

A confirmatory factor analysis of the classical HBM was made with data from the post-intervention survey by using Lisrel 8.70 [24], since the sample size of the survey was larger. The maximum likelihood estimation in the analysis and the factors were permitted to correlate. The indices used to assess goodness of fit for the model included the root-mean square error of approximation (RMSEA; values of 0.08 or less indicate good fit), the comparative fit index (CFI; values of 0.90 or greater indicate good fit), the Goodness of Fit Index (GFI; values of 0.90 or greater indicate good fit), the Adjusted Goodness of Fit Index (AGFI; values of 0.90 or greater indicate good fit), the Normed Fit Index (NFI; values of 0.90 or greater indicate good fit) and the Incremental Fit Index (IFI; values of 0.90 or greater indicate good fit) [24].

It was assumed that individuals who perceived more barriers to taking action would pay little attention to the cues to action. In order to test this, a path from BAR to CTA was added.

### Intervention evaluation

#### (1) Participants and sampling method

This was a community-based project; health educations for injury control were not only done within high schools but also primary schools and kindergartens. A stratified sampling was used to obtain respondents from high schools, primary schools and kindergartens. Because different questionnaires were used for children in high schools, primary schools and kindergartens, the effectiveness of health education was evaluated only for the high school students in this report. In the pre-intervention survey all 843 students in grade one and grade two of Dongchuan high school in Shanghai in 2009 were selected based on our sample size calculation and this size would be large enough to detect the health belief changes of interest. In the post intervention survey, all 1269 students in grade one and grade two of the same school in 2012, which had been extended, including 838 from main campus and 431 from new campus were selected. The pre-intervention survey included 400 (47.4%) boys and 443 (52.6%) girls 15 to 19 years of age (16.91 ± 0.81 years). The post-intervention survey included 679 (53.4%) boys and 590 (46.6%) girls 15 to 18 years (16.93 ± 0.78 years). There was no significant difference in age and gender distributions between the pre-intervention and post-intervention groups.

#### (2) Procedure and intervention

In November 2009, the pre-intervention survey on injury-related health belief was performed using the self-administrated questionnaire. The participants were fully informed concerning the aim and significance of the survey and their rights to withdraw and provided oral consents. The study received ethical permission from the ethical committee of Fudan University, China.

During the period of January 2010 to the end of 2011 various forms of injury-related health education activities (e.g. workshops on traffic safety, workshops on escaping fire, lectures on safe community, etc.) were carried out at the three levels including community, schools and families. At the school and community levels, workshop was the main form of health education supplemented by situation simulations like fire escape drill and first aid training. At the family level, parents lectures were the major form and parents were trained in health educations (see Table 2 for intervention information).

**Table 2:** Health education interventions for injury

**Level**	**Interventions**
**Community**	▪ Three traffic safety lectures provided by police department.
	▪ Two fire safety education seminars provided by fire department.
	▪ Community fire drill (fire safety and escape skill) during summer holidays.
	▪ Lectures on safe community.
	▪ One first-aid skills training session.
	▪ Traffic safety and legal education seminars organized by community committees.
	▪ One sports injury prevention seminar organized by community committees.
	▪ One military and explosion show.
	▪ One food safety education seminar organized by community committees.
**School**	▪ Improved school safety environment (sports equipment updating and anti- slip measures strengthening).
	▪ Safety education courses focusing on safety behavior and injury prevention.
	▪ Regular injury prevention training for teachers.
**Family**	▪ Pamphlets and leaflets about injury prevention distributed to household regularly.

Students were required to participate in these health education events unless there was any particular reason (e.g. illness, or other compelling factors). In addition, warning signs were set up for injury-prone environment in schoolyard, special equipment, special occasions and sources of danger. Schools also strengthened campus security management against campus violence. A post-intervention survey was carried out in February 2012, 6 weeks after the intervention, by using the same questionnaire for the pre-intervention survey.

#### (3) Comparison of health belief scores

Health belief scores were calculated for both pre-intervention and post-intervention using the CFAWS and the SSS methods, respectively. Estimated weight of each item (*W*_*n*_) in the CFAWS method was obtained from confirmatory factor analysis of the classical HBM.

➢ In the CFAWS method:

Wc (WHBS) = W_1_ × IS_1_ + W_2_ × IS_2_ + … + W_22_ × IS_22,_ where *W*_*n*_ (n = 1,2…22) was the estimated weight of each item, and *IS*_*n*_ (n = 1,2…22) was the initial score of each item.

➢ In the SSS method:

Health Belief Score (HBS) = IS1+ IS2 + … + IS22

Two independent sample t-tests were used to test the difference in health belief scores between pre-intervention and post-intervention. A significant level *p* = 0.05 was employed. Epidata 3.1 was used for data management; SPSS 18.0 was used for variable distribution and significance testing. Missing values were treated using listwise deletion method.

## Results

### Reliability test of the questionnaire

The result of the questionnaire reliability test showed that reliability coefficients (Cronbach’s alpha) ranged from 0.89 to 0.94 for the five parts (SUS, SER, BEN, BAR, CTA), and the average reliability coefficient was 0.89 for the 22 items.

### Results of CFA

**Figure 2:**
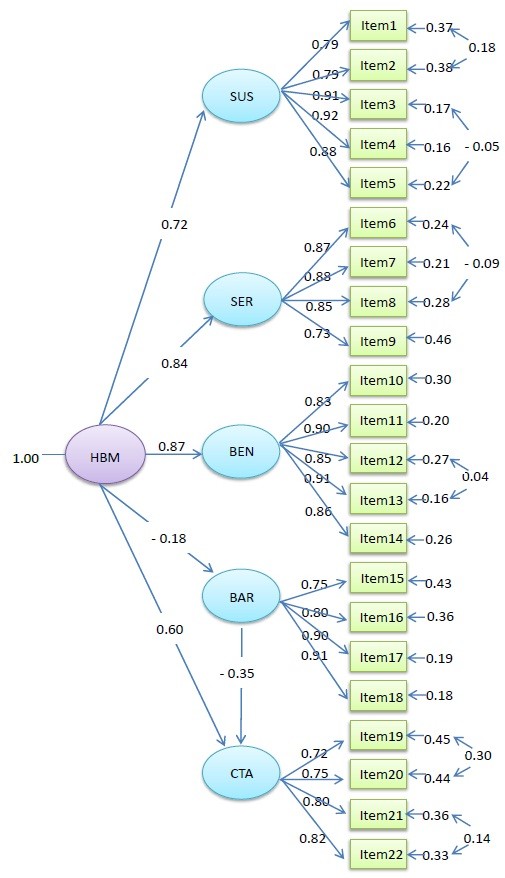
HBM path diagram.

The data fit the HBM well from the CFA (*p* < 0.001), the indices used to assess goodness of fit for the HBM were as follow: Chi-Square = 871.24 (df = 196), RMSEA = 0.041(90% confidence interval: 0.040, 0.045), GFI = 0.96, AGFI = 0.95, NFI = 0.98, CFI = 0.98, IFI = 0.98. The standardized path coefficients for five factors (SUS, SER, BEN, BAR and CTA) in the HBM were 0.72(*p* < 0.001), 0.84(*p* < 0.001), 0.87(*p* < 0.001), -0.18(*p* < 0.001) and 0.60(*p* < 0.001), respectively. The result of CFA showed that BEN and SER had the greatest impact on the health belief, SUS and CTA were the second and third most important components of HBM respectively. BAR had no notable effect. Though BAR had some impact on CTA, the standardized path coefficient was only 0.35. Figure 2 shows the factor structure of the HBM. Based on the CFA of the classical HBM, Table 1 shows the total effect (TE) of health belief on each item, TE represents the degree of the changes of observed variables when the health belief changes by one unit and it was the estimated weight (W_*n*_) of each item.

### Result of health belief scores comparison

Two independent sample t-tests of health belief scores showed that health belief scores were higher in the post-intervention than in the pre-intervention (*p* < 0.001) using either the CFAWS method or the SSS method. The mean differences was 1.64 (95% confidence interval: 2.33, 0.94) in CFAWS method and 2.97 (95% confidence interval: 3.99, 1.95) in SSS method, which represented improvement in community-based health education among high school students.

## Discussion

Health belief is important since it predicts health behavior. The HBM is useful because it not only helps understand health behavior but also guides interventions by identifying potentially modifiable antecedents of health behavior. The results of CFA provide further empirical support for the HBM. Since there are few studies in which CFA are used to explain and evaluate HBM, this study can be seen as a pilot study in this regard. CFA of the HBM gives indication that to improve the health belief of students the most important thing is to make them aware of the benefits of injury prevention action, and susceptibility and consequences of injury, and to enhance their perception of injury cues to action; BAR seems not important for health belief and does not lower students’ perception of CTA, notwithstanding this, efforts to remove or minimize all barriers to engaging in health behavior should continue. The comparison of the health belief score between pre-intervention and post-intervention using both the SSS method and the CFAWS method shows that the community-based health education programme for injury prevention presents a significant improvement. However, the difference is based on data from two cross-sectional surveys, the evidence is relatively weak regarding individual variations.

Another significant aspect of this study is that a weighted scoring method based on CFA, or the CFAWS method is used to calculate the health belief score. This allows researchers not only to test the reliability of the questionnaire but also verify the validity of the health belief model. The CFAWS method has the same advantages as the latent variable score model and is easy to be mastered by researchers and understood by readers. As for the SSS method, it does not account for the impacts of measurement errors and correlations among variables, and is not as precise as the CFAWS method. Therefore, the CFA weighted score method is preferred in the evaluation of the intervention programme.

One limitation is that the study involved one school and therefore the generalizability is limited. Another limitation is that different samples were used for pre- and post-intervention surveys. The proposed CFAWS method to evaluate the effectiveness of health education based injury-related interventions among high school students has not yet been validated in other settings. Even though we explore the CFAWS from a methodological aspect, we have not taken into consideration social environmental factors, injury-related risk behaviors and injury outcomes in the theoretical construction of the health belief model yet. In addition, since the post-intervention survey was conducted 6 weeks after all the intervention, the data may only suggest short-term improvements in health behavior as a result of the intervention. Since many different intervention efforts were carried out, it is difficult to identify which intervention exactly had the desired effect.

## Conclusion

The community-based health education programme showed a significant effect in improving high school students’ health belief, and this study used a new technique for the evaluation of injury-related health belief, however, as this is only a small study in one school, further large scale studies are recommended. School health promotion programmes are believed to be most effective when they are developmentally appropriate and when they take into account the relationships among student, family, school and community [25]. In Shanghai, schools are administrated by the Education Bureau of the municipal and district government. Many of the injury prevention projects are usually functioned within the education system with a weak linkage with community. In recent years, along with the International Safe Community Project [26,27], community and schools have been cooperating more frequently in injury prevention and safety promotion. The advantages of this cooperation are: (1) the community can coordinate multiple resources from, traffic departments, fire departments and health departments; the cooperation facilitates the community to highlight the specific type of injuries and risk factors and provide technological support to schools in executing injury prevention programmes. (2) With the help of the residential committee, the community can gain support from families. (3) The community has a special fund and can provide financial support for injury prevention projects. (4) The community can provide a necessary arena for the intervention. (5) Students can spread the knowledge and skills about injury prevention to families and communities.

Schools should be a secure environment where young people can develop their full potential; strengthening the role of schools as healthy environments that support the academic, social and emotional growth of students is essential. A concerted effort to improve school environments in a wide variety of communities can provide more resources and adequate funding. A long-term commitment by the general public, politicians and healthcare and educational systems therefore, is necessary to ensure positive outcomes from these community-based health education programmes.

## Competing interests

The authors declared that they have no conflict of interests.

## Authors’ contributions

Conceived and designed the experiments: WSM, CZJ, CY. Performed the experiments: CZJ, WSM. Analyzed the data: CZJ, CY, WSM. Contributed reagents/materials/analysis tools: WSM. Wrote the manuscript: CZJ, CY, WSM. Critically reviewed the paper: WSM, CY, CZJ. All authors read and approved the final manuscript.

## Pre-publication history

The pre-publication history for this paper can be accessed here:

http://www.biomedcentral.com/1471-2458/14/26/prepub
